# Splitting Forceps

**Published:** 1866-03

**Authors:** Gam’L Jackson

**Affiliations:** Winona, Minn.


### SPLITTING FORCEPS.

BY DR. GAM'L JACKSON, WINONA, MINN.

There is probably no other instrument in dental surgery quite so illy adapted to its purpose as the ordinary splitting forceps. This instrument is required in extracting the inferior molars under various circumstances; where there is exostosis, great divergence or convergence of the roots, or where, from whatever cause, the tooth appears to be "clinched under the jaw," and resists the usual efforts to dislodge it.

Not one dentist in twenty has a splitting forceps, and not one in fifty has ever used one. The chief reason of this is because the instrument is not properly constructed, rather than on account of the infrequency of cases requiring its use.

Within the past two years I have had four cases of first and second molars which required splitting, besides being the subject of another case myself. The first and second efforts, made by a strong and skillful practitioner, in the latter case, resulted in failure. The third effort, by my own direction to use steady lateral force, accomplished the evulsion of the tooth, at the expense of the external alveolar plate, reaching to the adjacent teeth.

Of the other four cases, I succeeded in luxating the teeth in two instances, the others remaining firm. Three of these teeth I left in the jaw, but by splitting the other one, which, but for this operation, would also have remained, its roots were removed without difficulty. In this and my own case, the roots were bulbous.

All of these teeth were in unbroken arches ; and as they all had full crowns, the probable result of an application of the separating forceps, as they are now made, would have been a general "loosening up" of at least all the teeth posterior to the one being split.

The splitting forceps of the depots are defective in the following respects : power, strength and shape. To be efficient and safe, they should have double their present leverage ; they should be as nearly inflexible as possible ; the cutting edge of the blades should have curve enough to insure the fracture through the bifurcation ; and, most important of all, they should be provided with a perfectly reliable, peculiar stop, to prevent the closing of the blades further than necessary to split the tooth.

With such an instrument any inferior molar might be split, in a crowded arch, without danger to the contiguous teeth, and the operation of extraction be made easier to both patient and operator.

